# Muscle shear wave elastography in idiopathic inflammatory myopathies: a case–control study with MRI correlation

**DOI:** 10.1007/s00256-019-03175-3

**Published:** 2019-02-27

**Authors:** Abdulrahman M. Alfuraih, Philip O’Connor, Ai Lyn Tan, Elizabeth M. A. Hensor, Andreas Ladas, Paul Emery, Richard J. Wakefield

**Affiliations:** 1 Radiology and Medical Imaging Department, College of Applied Medical Sciences, Prince Sattam bin Abdulaziz University, Kharj, Saudi Arabia; 2 Leeds Institute of Rheumatic and Musculoskeletal Medicine, 2nd Floor, Chapel Allerton Hospital, University of Leeds, Chapeltown Road, Leeds, LS7 4SA UK; 3 NIHR Leeds Biomedical Research Centre, Leeds Teaching Hospitals NHS Trust, Leeds, UK

**Keywords:** Shear wave elastography, Elastography, Muscle, Myositis, Ultrasound

## Abstract

**Objective:**

To investigate muscle stiffness in patients with idiopathic inflammatory myopathies (IIM) using shear wave elastography (SWE) and to correlate the results with muscle strength and MRI features of myositis.

**Materials and methods:**

Muscle shear wave velocity (SWV) was measured in 23 active IIM patients (13 females, mean age 50.4 ± 16.1 years) and 23 matched healthy controls (13 females, mean age 50.7 ± 16.2 years). The investigated muscles included the vastus lateralis (VL), rectus femoris (RF), vastus medialis (VM) vastus intermedius (VI), biceps femoris (BF), semitendinosus (ST), semimembranosus (SM) and the biceps brachii (BB) scanned during relaxed resting and passive stretching positions. Participants performed multiple tests to evaluate their muscle strength. IIM patients had a thigh MRI to assess degrees of oedema, fatty infiltration and atrophy.

**Results:**

In the resting position, IIM patients had a 12.9–22.2% significantly lower SWV (*p* < 0.05) for the quadriceps and hamstrings, but not BB. There was no difference during passive stretching. The SWV for VL, VI and BF showed moderate correlations with the muscle strength tests ranging from *r* = 0.47 to *r* = 0.70 (all *p* < 0.05). Lower SWV was associated with greater MRI scores of oedema (*p* = 0.001) and atrophy (*p* = 0.006). However, SWV did not correlate with fatty infiltration (*r* < 0.3; *p* = 0.28), creatine kinase (*r* = 0.28; *p* = 0.19) or disease duration (*r* = 0.26; *p* = 0.24).

**Conclusion:**

Shear wave elastography may detect abnormal reduced thigh stiffness in IIM patients. SWE measurements were significantly associated with muscle weakness and MRI signs of oedema and atrophy. Future research should investigate this new technology for monitoring disease activity.

**Electronic supplementary material:**

The online version of this article (10.1007/s00256-019-03175-3) contains supplementary material, which is available to authorized users

## Introduction

Idiopathic inflammatory myopathies (IIM) are a group of systemic autoimmune inflammatory muscle disorders characterised by muscle weakness, inflammation and structural changes [1]. The early identification of these patients is important as they may often have a poor prognosis and quality of life [2]. Although clinical history and physical examination remain an important aspect of disease assessment, other methods are often required to improve diagnostic certainty. However, such additional assessment methods have several limitations; invasiveness (muscle biopsy), poor sensitivity and specificity (serology for muscle enzymes and electromyography) or cost and availability (MRI) [3].

Shear wave elastography (SWE) is a relatively new ultrasound-based technique that has shown promise for the investigation of musculoskeletal disorders such as tendinopathy [4–6], muscle spasticity [7, 8] and Duchenne muscular dystrophy [9, 10]. However, relatively little work has focused on muscle. In contrast to the older type of sonoelastography known as strain elastography, SWE has the advantage of providing a quantitative measure of tissue stiffness. A methodology for evaluating SWE in normal muscle has recently been published [11, 12]. We hypothesize that SWE could potentially be helpful for the investigation of muscles of patients with IIM, as the presence of inflammatory cells and exudate, thickened capillaries, peri-fascicular atrophy and myofiber necrosis [13] may result in an alteration of the mechanical properties of the tissue.

There are limited published data evaluating muscle elasticity in patients with IIM. A previous study used magnetic resonance elastography (MRE) [14] and reported a significantly decreased muscle stiffness. In contrast, strain elastography studies [15–17] reported increased muscle stiffness [16] and correlations with muscle enzymes [15] and echogenicity [17]. However, these studies are difficult to compare owing to their varying design and methodologies. All lacked any quantification of intrinsic muscle elasticity and comparison with a healthy control group. The quantitative characteristic of muscle elasticity in IIM is not well understood.

The primary objective of this study was to establish the face validity of muscle SWE by examining the quantitative characteristics of muscle elasticity in IIM patients compared with healthy controls. The secondary objective was to investigate the criterion validity of muscle SWE by correlating it with MRI-reported features of muscle oedema, atrophy and fatty infiltration, and with measures of muscle strength, serum creatine kinase level and disease duration.

## Materials and methods

### Study design

The study was conducted prospectively as a case–control study. It was approved by the local research ethics committee and all participants provided written informed consent. Recruitment began in May 2017 and ended in July 2018. Myositis patients were recruited from the outpatient rheumatology clinics and inpatient wards at Leeds Teaching Hospitals Trust. Sex and age frequency-matched healthy controls (matching the number of patients within categories defined by sex and 5-year age bands) were recruited from the University of Leeds and the Leeds Teaching Hospitals Trust and via advertisements on social media. No formal sample size/power calculations were carried out owing to a lack of available data. However, to estimate parameters for powering future clinical trials, published rules of thumb recommend between 12 and 30 subjects per group of interest [18, 19].

### Inclusion/exclusion criteria

For IIM patients, we recruited adult patients with an established diagnosis of adult IIM according to previously described criteria [20, 21]. Additionally, we only included patients with active disease who presented demonstrable muscle weakness (determined subjectively by a qualified physician or quantitively by a manual muscle test–8 score < 125/150) and at least two abnormal measures from the following:

1. Elevation of serum creatine kinase (CK) at a minimum level of 1.3 times the upper limit of normal
2. Patient global visual analogue scale (VAS) score > 20 mm/100 mm
3. Physician global VAS score > 20 mm/100 mm [22]

Patients were excluded if they had a history of spinal disease or neuropathy, or any contraindication to MRI. For healthy controls, we recruited asymptomatic adults (aged > 18 years) with no previous history of muscle disorders, arthritis or neuropathy. None of the participants was currently on HMG-CoA reductase inhibitors (statins).

### Demographic and clinical information

Basic information was collected, including age, sex, BMI, alcohol consumption, smoking and global VAS score. Additionally, subtype of myositis, disease duration and current medications were recorded for IIM patients. The latest CK level reported within the last month was documented.

### Shear wave elastography

The SWE system employed was the Aixplorer (Supersonic Imagine, Aix-en-Provence, France) system using the SuperLinear™ SL10-2 MHz probe, which has demonstrated substantial reliability in muscle SWE [12]. The muscles investigated were the four quadriceps (vastus lateralis [VL], rectus femoris [RF], vastus medialis [VM] and vastus intermedius VI]), the three hamstrings (biceps femoris [BF], semitendinosus [ST] and semimembranosus [SM]) and the biceps brachii (BB) of the most symptomatic side in patients and the dominant side in controls. These muscles were chosen as proximal lower and upper limb muscles, which are known to be commonly affected in IIM [20]. Positioning was as follows: resting supine on a flat bed with knees on full extension (rested quadriceps); elbow flexed at 90° with the forearm rested on the body and hand in supination (biceps brachii); prone on a flat bed with the knees flexed at 90° and rested on a wall (hamstrings). The quadriceps were also tested under passive stretching with the participants seated while the hips and knees were flexed at 90° without touching the floor. This was tested to evaluate if IIM muscle stiffness behaves differently under the passive load acted upon by the limb weight. Participants were instructed to relax their muscles in all positions and were rested supine for 5 min before scanning.

Two-dimensional SWE acquisitions were repeated three times per muscle and recorded as shear wave velocity (SWV) in units of metres per second (m/s). The probe was oriented along the muscle fibres and placed on top of the skin with a minimal load while ensuring no external pressure could deform the tissues and affect the measurements. The operator was blinded to the MRI and muscle assessment results.

### Muscle assessment

All participants performed four muscle assessment tests. First, handgrip strength was assessed using the Jamar Plus+® electronic hand dynamometer (Lafayette Instrument Company, Lafayette, LA, USA) using established protocols [23]. Next, participants performed the expanded timed get-up-and-go test (ETGUGT) to assess essential functional tasks such as standing, balancing and walking [24].

The 30-s chair stand test (CST) was performed afterwards to test lower body strength and endurance, which has good responsiveness and construct validity in IIM [25, 26]. Lastly, isokinetic knee extension/flexion strength was tested using the Biodex system 4 (IRPS Mediquipe, UK). Participants performed three sets of maximum effort concentric knee extensions and flexions at an angular velocity of 60°/se to calculate the peak torque (Newton metres [Nm]) and average power (Watts) based on a mean of three repeated sets [27]. These strength and power measures were normalised to body weight (Nm/kg and W/kg).

### Magnetic resonance imaging

All IIM patients had a thigh MRI scan using the Siemens Magnetom Verio 3.0 T scanner (Siemens Healthcare, Germany) powered by the syngo (MR B17) software. Axial, coronal and sagittal images were acquired of the same side using the MRI protocol described in Table 1 of the supplementary material.

**Table 1:** Clinical data of all idiopathic inflammatory myopathies (*IIM*) patients

Case number	Sex	Age (years)	Diagnosis	Disease duration (months)	CK level (IU/L)^a^	Treatment
1	Female	76.5	Undifferentiated IIM	20	601	Prednisolone
2	Male	54.7	Undifferentiated IIM	1.1	708	Methylprednisolone, prednisolone
3	Female	58.6	Overlap myositis (polymyositis and rheumatoid arthritis)	0.2	2,662	Methylprednisolone, prednisolone
4	Female	63.0	Undifferentiated IIM	6.5	324	Methotrexate, prednisolone
5	Male	38.2	Dermatomyositis	0.5	1,375	None
6	Female	50.9	Polymyositis	2.4	1,000	Mycophenolate, prednisolone
7	Male	57.0	Polymyositis	108.7	757	Methotrexate
8	Male	40.7	Undifferentiated IIM	34.8	12,802	Methotrexate, prednisolone
9	Female	74.6	Undifferentiated IIM	2.1	777	Intravenous immunoglobulins, prednisolone
10	Female	35.8	Polymyositis	8.1	1,205	Prednisolone, azathioprine
11	Female	59.1	Polymyositis	198.5	347	Methotrexate
12	Male	77.9	Inclusion body myositis	6.1	190	Methotrexate
13	Female	23.1	Dermatomyositis	0.4	4,553	Prednisolone
14	Female	40.5	Dermatomyositis	8.6	70	Hydroxychloroquine
15	Male	52.7	Overlap myositis (mixed connective tissue disease and myositis)	64.3	692	Methotrexate, hydroxychloroquine
16	Female	49.1	Dermatomyositis	177.2	1,784	Mycophenolate, hydroxychloroquine, prednisolone
17	Male	58.4	Inclusion body myositis	19.9	399	Methotrexate
18	Female	43.6	Undifferentiated IIM	1.6	763	Methotrexate, hydroxychloroquine
19	Female	53.5	Undifferentiated IIM	26.3	72	Mycophenolate, prednisolone
20	Male	49.8	Polymyositis	44.9	1,225	Cyclophosphamide, prednisolone
21	Female	21.0	Undifferentiated IIM	31.2	33	Hydroxychloroquine
22	Male	60.5	Inclusion body myositis	0.4	1,184	None
23	Male	20.4	Polymyositis	2.1	634	Prednisolone

^a^Normal value is 25–200 IU/L for women and 40–320 IU/L for men

The images of the quadriceps and hamstrings muscles were scored based on three main aspects: muscle oedema, fatty infiltration and muscle atrophy based on previously described methods [28–30]. Each muscle was scored as normal, mild, moderate or severe for each feature according to the definitions and scoring criteria explained in the supplementary material (Table 2). This semi-quantitative scoring was performed by two musculoskeletal radiologists experienced in reading muscle MRI (25 and 11 years of experience). The radiologists were only aware that they were scoring IIM patients, but were blinded to other clinical, laboratory, SWE and muscle strength information. Inter-reader reproducibility was analysed to assess scoring agreement. Post-individual scoring, cases of disagreement between the radiologists were reconciled by consensus.

**Table 2:** Characteristics of the study participants

Characteristic	IIM patients		Healthy controls		Difference (%)	95% CI of the difference	*p* value*
	Mean (%)^a^	95% CI	Mean (%)^a^	95% CI			
Sex	13 females (56.5)	–	13 females (56.5)	–	–	–	1.00
Age	50.4 (16.1)	43.4, 57.4	50.7 (16.2)	43.7, 57.7	−0.31 (−0.6)	−9.9, 9.3	0.95
Males	51.0 (15.4)	40.0, 62.0	52.1 (16.0)	40.7, 63.6	−1.1 (−2.1)	−15.8, 13.6	0.87
Females	49.9 (17.2)	39.6, 60.3	49.6 (17.0)	39.4, 59.9	0.3 (0.6)	−13.5, 14.1	0.96
Height (cm)	169.0 (9.8)	164.7, 173.4	169.3 (10.5)	164.7, 173.8	−0.3 (−0.2)	−6.3, 5.8	0.92
Weight (kg)	75.2 (11.2)	70.1, 80.2	72.9 (14.4)	67.0, 79.1	2.25 (3.2)	−5.5, 10.0	0.56
Body mass index	26.5 (5.4)	24.2, 28.8	25.3 (3.9)	23.6, 26.9	1.25 (4.7)	−1.5, 4.0	0.37
Waist–hip ratio	0.90 (0.01)	0.86, 0.94	0.86 (0.01)	0.82, 0.90	0.04 (4.7)	−0.01, 0.09	0.16
Smoking	9 (39)	–	12 (52)	–	–	–	0.37
Smoking pack-years	21.0 (15.6)	9.7, 33.7	13.1 (19.6)	0.7, 25.6	8.6 (60.3)	−8.0, 25.2	0.29
Drinking alcohol	8 (35)	–	5 (22)	–	–	–	0.33
Consumption (units/week)	3.7 (3.5)	0.7, 6.7	7.5 (2.5)	4.3, 10.7	−3.8 (−50.7)	−7.9, 0.2	0.06
Visual analogue score (mm)	53.2 (19.0)	45.0, 61.4	11.3 (16.0)	4.4, 18.3	41.8 (370)	31.4, 52.3	*<0.001*
ETGUGT, sit to stand (s)	2.5 (3.0)	1.0, 3.9	1.0 (0.3)	0.9, 1.1	1.5 (150)	0.2, 2.7	*<0.001*
ETGUGT, gait initiation (s)	1.4 (0.9)	1.0, 1.9	0.9 (0.4)	0.7, 1.0	0.6 (55.6)	0.1, 1.0	*<0.001*
ETGUGT, walk 1 (s)	6.2 (1.8)	5.4, 7.1	4.3 (0.8)	3.9, 4.7	1.9 (44.2)	1.1, 2.8	*<0.001*
ETGUGT, turn around (s)	4.3 (1.4)	3.6, 4.9	3.0 (0.5)	2.8, 3.2	1.2 (43.3)	0.5, 2.0	*0.002*
ETGUGT, walk 2 (s)	6.4 (1.9)	5.4, 7.4	4.4 (0.8)	4.1, 4.7	2 (45.5)	1.0, 3.0	*<0.001*
ETGUGT, slow, stop (s)	4.5 (1.7)	3.7, 5.3	2.7 (0.6)	2.4, 2.9	1.8 (66.7)	0.9, 2.6	*<0.001*
ETGUGT, total time (s)	25.3 (9.0)	20.8, 29.8	16.2 (2.7)	15.1, 17.4	9.1 (56.2)	4.4, 13.7	*<0.001*
30-s chair sit-to-stands	5.1 (5.4)	2.8, 7.5	18.6 (5.2)	16.3, 20.8	−13.4 (−72.6)	−16.6-10.3	*<0.001*
Handgrip strength (kg)	16.3 (10.4)	11.8, 20.8	37.6 (12.5)	32.0, 43.2	−21.3 (−56.6)	−28.2-14.4	*<0.001*
Knee extension torque (Nm/kg)	0.52 (1.02)^b^	0.29, 0.85	1.53 (0.70)^b^	1.25, 1.75	−1.01 (−66.0)	−1.22, −0.53	*<0.001*
Knee flexion torque (Nm/kg)	0.36 (0.28)^b^	0.24, 0.45	0.86 (0.43)^b^	0.70, 0.99	−0.5 (−58.1)	−0.64, −0.29	*<0.001*
Knee extension power (W/kg)	0.20 (0.62)^b^	0.06, 0.55	0.92 (0.43)^b^	0.72, 1.09	−0.72 (−78.3)	−0.82, −0.38	*<0.001*
Knee flexion power (W/kg)	0.17 (0.21)^b^	0.10, 0.26	0.55 (0.21)^b^	0.47, 0.63	−0.38 (−69.1)	−0.46, −0.24	*<0.001*

*ETGUGT* expanded timed get-up-and-go test

*p values significant at 95% are in italics. Continuous variables were tested via independent *t* test or Mann–Whitney *U* test, and categorical data were tested using the Chi-squared test

^a^Data in parentheses represent standard deviations for means or percentages for ratio

^b^Median and interquartile range (95% confidence interval for the median values is generated based on 1,000 bootstrap samples)

### Statistical analysis

Data analysis was performed using SPSS version 25 (IBM, Armonk, NY, USA) and GraphPad Prism version 7.00 (GraphPad Software, La Jolla, CA, USA). Descriptive analysis was performed to report the main characteristics of each muscle elasticity and was graphically represented in box plots. Intra-operator reproducibility of the repeated SWE measurements was analysed using intraclass correlations (ICC). Inter-reader agreement over the ordinal MRI scores was analysed using quadratic weighted Kappa coefficients (Kw).

Independent sample Student’s *t* test was used to determine if there was a significant difference in mean SWV values between patients and healthy controls per muscle. A receiver operating characteristic (ROC) curve and the area under ROC (AUROC) were reported to evaluate the ability of SWE to discriminate participants with and without IIM. Spearman’s correlation coefficients were calculated to correlate SWV with CK levels, disease duration in addition to the results of the muscle tests.

The association between SWE and MRI scores was evaluated using the Jonckheere–Terpstra test to detect significant monotonic trends between SWV and MRI scores. Kendall’s tau-b correlation coefficients were also calculated for the two variables. The 95% confidence intervals (CIs) were calculated where appropriate. All tests were two-tailed. Absolute correlation coefficients ≥ 0.3 and statistical significance at *p* < 0.05 was considered a potential effect worthy of further fully-powered investigation.

## Results

### Patients and characteristics

A total of 23 patients diagnosed with IIM volunteered to participate in this study (10 males and 13 females; mean age [SD] = 50.4 years [16.1]). Clinical data of the recruited IIM patients are listed in Table 1. With regard to disease activity, 4 patients had a normal level of CK, but re-presented with significant muscle weakness and were determined as clinically active by the physician. The patients were frequency-matched to 23 healthy controls. The descriptive characteristics of the patients and healthy controls are represented and tested for differences in Table 2. Five IIM patients failed to perform the ETGUG test, of 2 of whom also failed to perform the knee extension/flexion due to severe muscle weakness.

### Muscle shear wave elastography

**Fig. 1:**
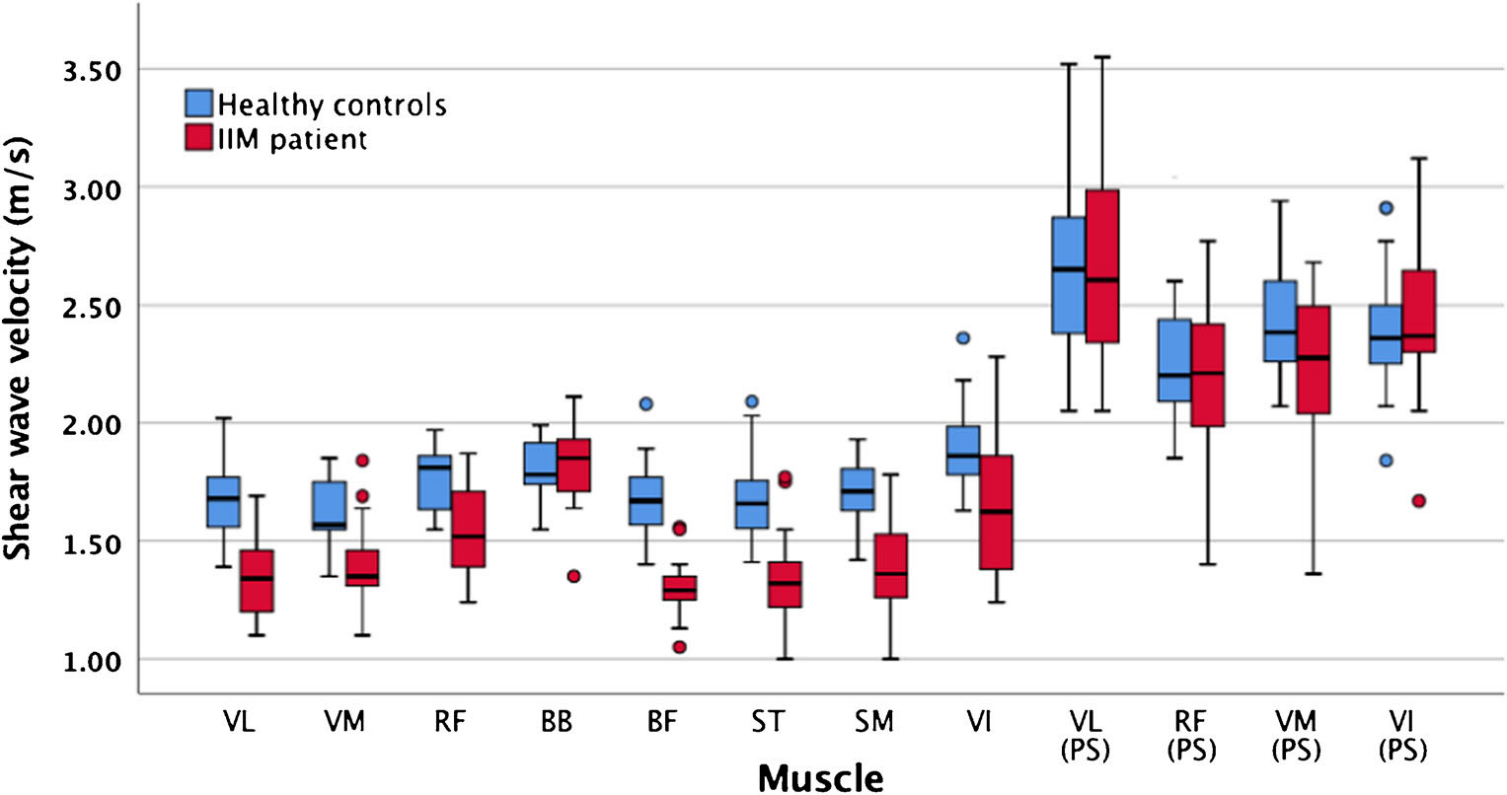
Clustered boxplot of shear wave velocity (m/s) by participant type. *VL* vastus lateralis, *RF* rectus femoris, *VM* vastus medialis, *VI* vastus intermedius, *BF* biceps femoris, *ST* semitendinosus, *SM* semimembranosus, *BB* biceps brachii, *PS* passively stretched

**Fig. 2:**
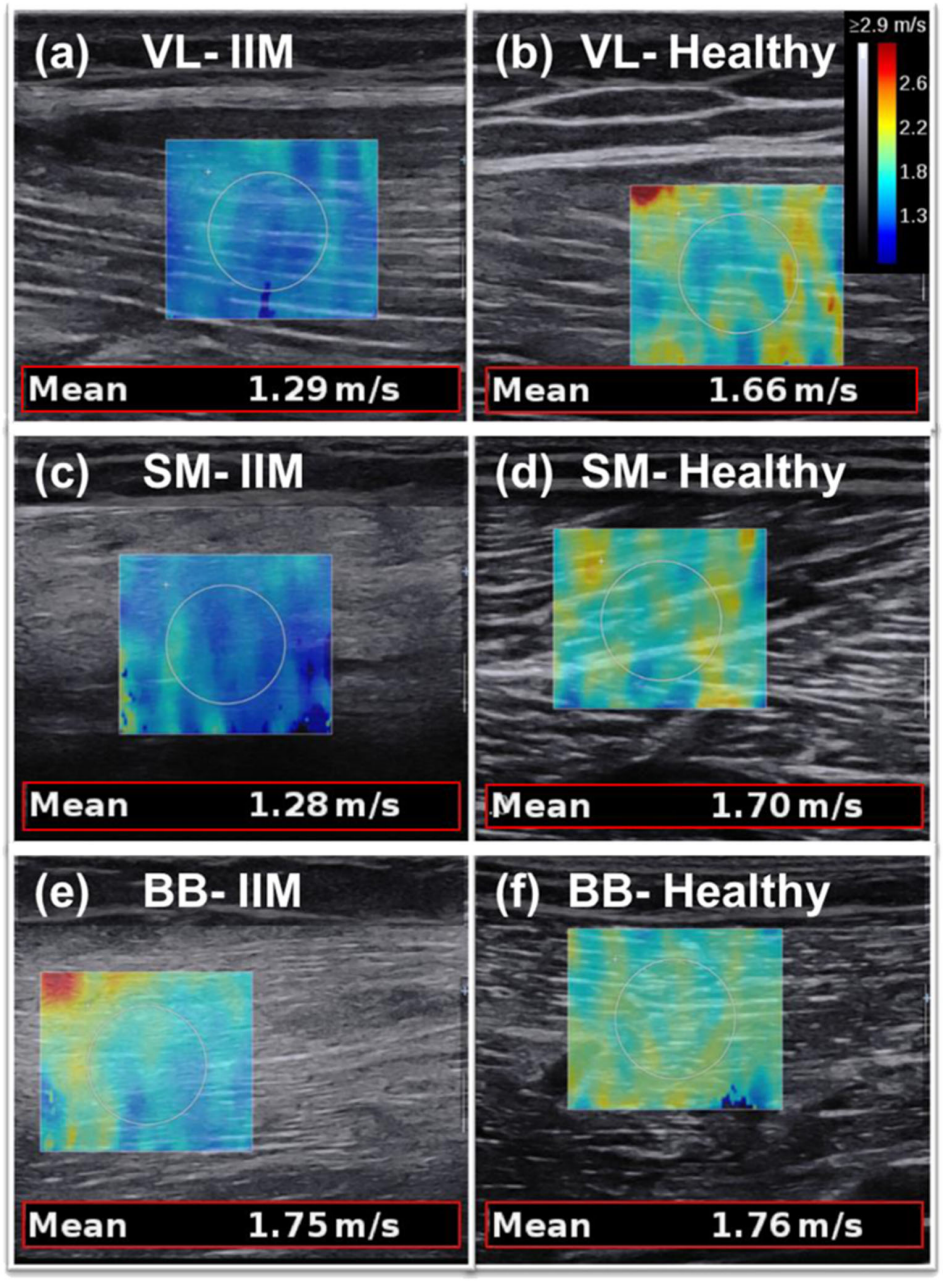
Shear wave elastography images from three idiopathy inflammatory myopathy (*IIM*) patients compared with comparable healthy controls’ muscles. *VL* vastus lateralis, *SM* semimembranosus, *BB* biceps brachii

The descriptive data for the SWV measurements are reported in Table 3. It shows the results of the independent sample t-test, which demonstrated a significantly lower muscle stiffness in IIM across all the quadriceps and hamstrings in the resting position (*p* < 0.05). In contrast, there was no significant difference for the BB or the quadriceps during passive stretching. The clustered boxplots in Fig. 1 graphically represent the SWV results for the various muscles of the two groups. Examples of SWE images from IIM patients and comparable controls are shown in Fig. 2. Sample size was too small to meaningfully compare SWV between IIM subtypes. Nevertheless, the inclusion body myositis patients showed no significant SWV difference from the other IIM patients (*p* = 0.24).

**Table 3:** Shear wave elastography measurements of the scanned muscles for the IIM patients and healthy controls

Muscle	IIM patients		Healthy controls		Difference	95% CI of the difference^b^	*p* value*	Effect size
	Median^a^	95% CI	Median^a^	95% CI				
Vastus lateralis	1.35 (0.32)	1.26, 1.44	1.68 (0.23)	1.62, 1.76	−0.33 (−19.6%)	−0.42, −0.20	*<0.001*	1.18
Passively stretched	2.60 (0.68)	2.36, 2.93	2.65 (0.51)	2.56, 2.81	−0.05 (−1.9%)	−0.31, 0.21	0.504	0.08
Rectus femoris	1.52 (0.33)	1.43, 1.65	1.81 (0.23)	1.71, 1.85	−0.29 (−16.0%)	−0.32, −0.10	*0.006*	1.02
Passively stretched	2.23 (0.53)	2.05, 2.43	2.20 (0.36)	2.12, 2.27	0.03 (1.4%)	−0.20, 0.21	0.989	0.06
Vastus medialis	1.36 (0.16)	1.33, 1.46	1.60 (0.21)	1.55, 1.74	−0.24 (−15.0%)	−0.34, −0.13	*0.002*	1.28
Passively stretched	2.28 (0.49)	2.10, 2.48	2.39 (0.38)	2.29, 2.58	−0.11 (4.6%)	−0.39, 0.03	0.091	0.25
Vastus intermedius	1.62 (0.49)	1.46, 1.82	1.86 (0.22)	1.78, 1.95	−0.24 (−12.9%)	−0.42, −0.08	*0.038*	0.63
Passively stretched	2.37 (0.39)	2.30, 2.56	2.36 (0.28)	2.25, 2.47	0.01 (0.4%)	−0.12, 0.23	0.454	0.02
Biceps brachii	1.85 (0.28)	1.72, 1.90	1.78 (0.20)	1.75, 1.90	0.07 (3.9%)	−0.08, 0.14	0.509	0.28
Biceps femoris	1.30 (0.14)	1.28, 1.45	1.67 (0.20)	1.58, 1.76	−0.37 (−22.2%)	−0.44, −0.26	*<0.001*	2.14
Semitendinosus	1.33 (0.31)	1.26, 1.40	1.66 (0.23)	1.58, 1.70	−0.33 (−19.9%)	−0.42, −0.187	*0.001*	1.21
Semimembranosus	1.36 (0.28)	1.28, 1.51	1.71 (0.18)	1.63, 1.76	−0.35 (−20.5%)	−0.44, −0.23	*<0.001*	1.48

*p values significant at 95% are in italics. Results are based on independent sample *t* test of natural log-transformed values

^a^Data in m/s with interquartile range (95% confidence interval for the median values are generated based on 1,000 bootstrap samples)

^b^The 95% confidence intervals (*CIs*) for the difference between the medians is calculated based the Hodges–Lehmann method [31]

**Fig. 3:**
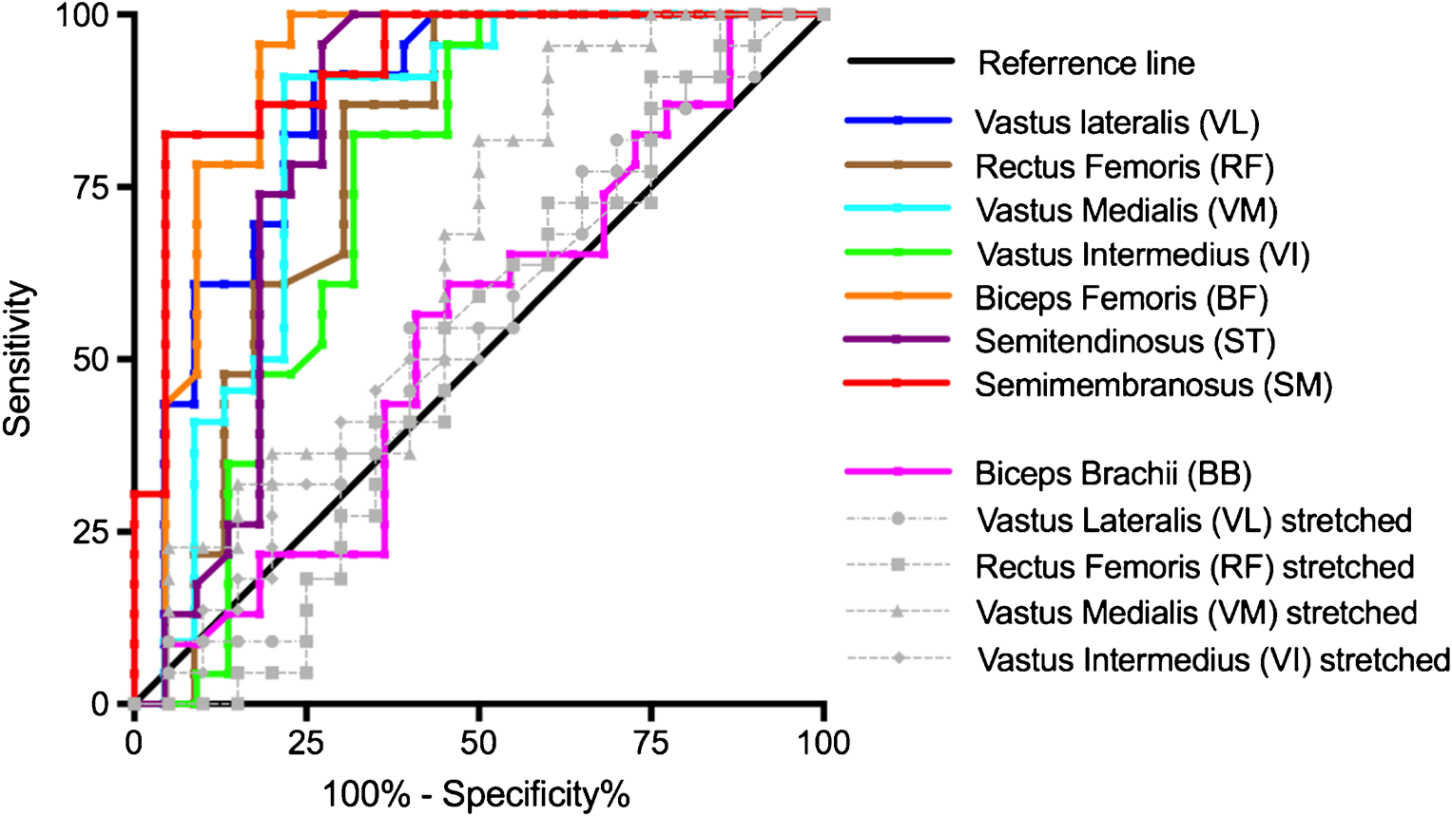
Receiver operating characteristic curve for shear wave elastography performance in discriminating IIM and healthy muscles

Intra-operator reproducibility showed substantial reliability with ICC > 0.95 for all muscles except for the stretched RF, which had a slightly lower ICC of 0.84 (Supplementary Table 3). ROC curve for SWE was plotted in Fig. 3 using clinical diagnosis (based on a composite of clinical assessments and investigations) as the reference standard. SWE had AUROC results demonstrating an excellent level of discrimination for the quadriceps and hamstrings in the resting position (Table 4).

**Table 4:** Area under the operating characteristic results for the muscles tested using shear wave elastography based on disease presence

Muscle	AUROC	95% CI	*p* value*
Vastus lateralis	0.865	0.754, 0.975	*<0.001*
Passively stretched	0.525	0.344, 0.705	0.782
Rectus femoris	0.790	0.649, 0.930	*<0.001*
Passively stretched	0.507	0.324, 0.689	0.940
Vastus medialis	0.822	0.689, 0.954	*<0.001*
Passively stretched	0.644	0.471, 0.817	0.110
Vastus intermedius	0.746	0.590, 0.901	*0.005*
Passively stretched	0.548	0.370, 0.725	0.560
Biceps brachii	0.532	0.359, 0.704	0.717
Biceps femoris	0.908	0.806, 1.000	*<0.001*
Semitendinosus	0.822	0.681, 0.963	*<0.001*
Semimembranosus	0.925	0.846, 1.000	*<0.001*

*Significant *p* values are in italics

### Shear wave elastography correlations with clinical variables and muscle tests

Shear wave elastography correlations with CK and disease duration were generally weak (r_s_ = 0.28 [*p* = 0.19] and r_s_ = 0.26 [*p* = 0.24] respectively). However, slower walking speeds in the ETGUG were associated with lower SWV for the VL (r_s_ = −0.56), VI (r_s_ = −0.64) and RF (r_s_ = −0.51). Stronger grip strength was associated with higher SWV for the VL (r_s_ = 0.47), BF (r_s_ = 0.62) and SM (r_s_ = 0.45). A higher number of chair stands correlated positively with SWV for VL (r_s_ = 0.51, *p* = 0.012). The knee strength test correlated only with the BF; the correlations were explicably stronger for the flexion movement (peak torque r_s_ = 0.60, power r_s_ = 0.53) compared with the extension movement (peak torque r_s_ = 0.47, power r_s_ = 0.48). The full correlation results are provided in Supplementary Table 4.

### Magnetic resonance imaging

**Fig. 4:**
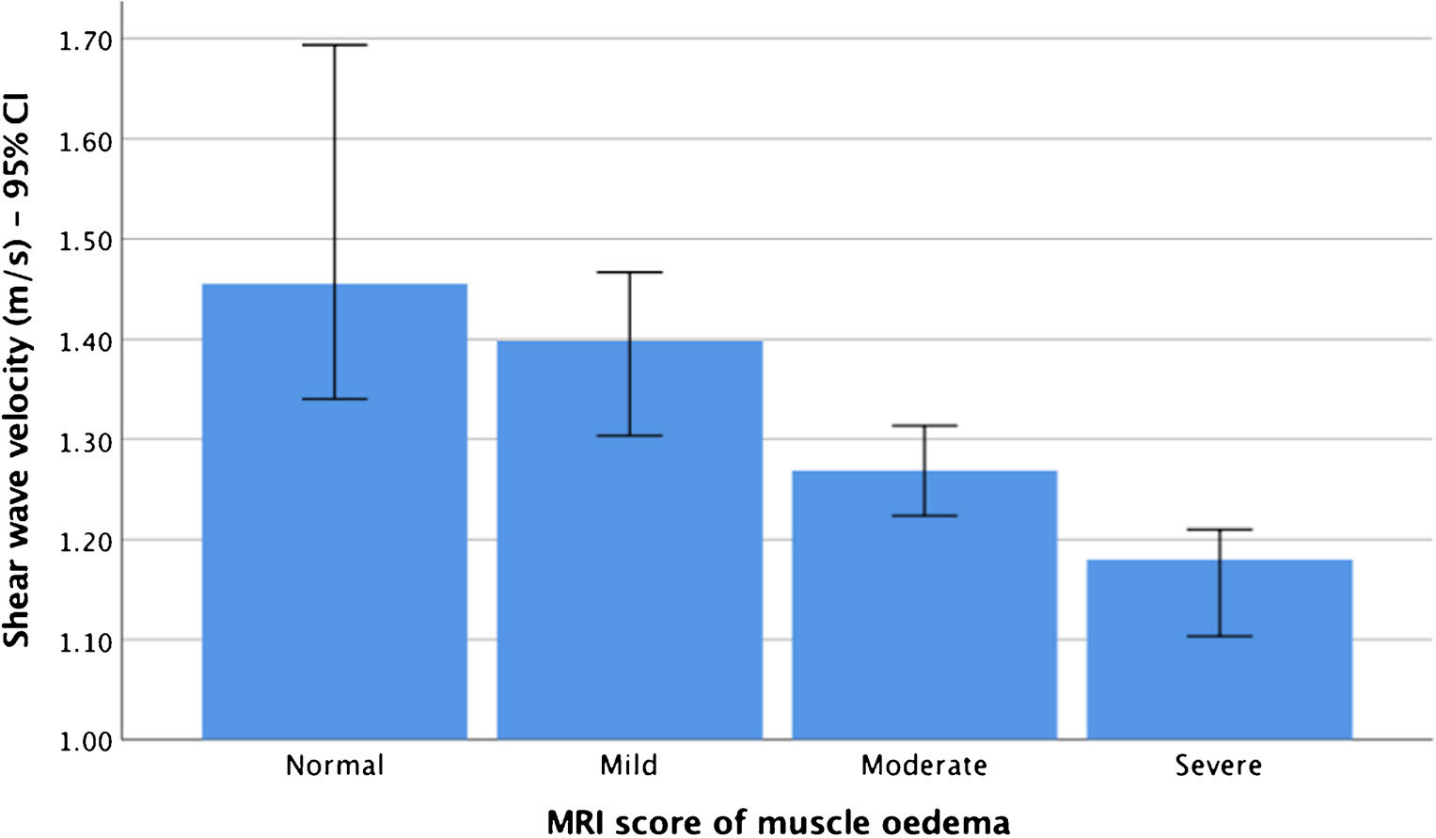
Bar graph of the significant decreasing monotonic trend between muscle stiffness and MRI oedema in the vastus medialis

Inter-reader agreement between the two radiologists was “almost perfect” for the overall MRI scoring of oedema, fatty infiltration and atrophy, with a weighted Kappa (95% CI) of 0.88 (0.83, 0.94), 0.88 (0.83, 0.93) and 0.83 (0.76, 0.90) respectively. The MRI results for the consensus scores are presented in Supplementary Table 5. The results of the consensus scores on the Jonckheere–Terpstra test and Kendall’s tau-b correlations for the associations between SWV and MRI were analogous (Table 5). They determined a statistically significant decreasing trend and a moderate negative correlation for SWV with oedema and atrophy in several muscles. In other words, higher MRI scores for oedema and atrophy were associated with lower SWV results. Figure 4 displays an example of the decreasing SWV scores with higher muscle oedema. However, the correlations between SWV and fatty infiltration across all muscles were weak (*r* < 0.3; *p* = 0.28).

**Table 5:** The association between MRI and shear wave elastography in IIM patients

Muscle		Oedema	Fatty infiltration	Atrophy
Vastus lateralis	Monotonic trend, *p* value*	*0.008*	0.27	0.14
	Correlation (*p* value)**	−0.405**** (0.008)	−0.099 (0.28)	−0.181 (0.14)
Rectus femoris	Monotonic trend, *p* value	0.26	0.42	0.33
	Correlation (*p* value)	0.112 (0.26)	−0.036 (0.42)	0.075 (0.33)
Vastus medialis	Monotonic trend, *p* value	*<0.001*	0.44	0.19
	Correlation (*p* value)	−0.553**** (0.001)	−0.019 (0.45)	−0.148 (0.19)
Vastus intermedius	Monotonic trend, *p* value	0.40	0.07	0.32
	Correlation (*p* value)	−0.042 (0.40)	−0.252 (0.07)	−0.083 (0.32)
Biceps femoris	Monotonic trend, *p* value	*<0.001*	0.054	0.20
	Correlation (*p* value)	−0.489**** (0.002)	−0.288 (0.054)	−0.150 (0.20)
Semitendinosus	Monotonic trend, *p* value	0.45	0.21	*0.037*
	Correlation (*p* value)	0.022 (0.45)	−0.137 (0.21)	−0.312*** (0.038)
Semimembranosus	Monotonic trend, *p* value	0.10	0.11	*0.006*
	Correlation (*p* value)	−0.219 (0.10)	−0.212 (0.11)	−0.444**** (0.006)

Significant *p* values are in italics

*The *p* value (one-sided test) of the Jonkheere–Terpstra test *p* value (significant at *p* < 0.05)

**The correlation coefficient and *p* value of Kendall’s tau-b correlations (significant at *p* < 0.05)

***Correlation is significant at the 0.05 level (two-tailed)

****Correlation is significant at the 0.01 level (two-tailed)

## Discussion

To our knowledge, this is the first case–control study to investigate muscle elasticity using SWE in IIM patients, and also the first to study the association of SWE with muscle strength and MRI features. The results, particularly the lower muscle stiffness measurements in IIM during the resting position across the lower limb muscles tested, support the face validity of SWE. The pathological mechanisms for this include the presence of destructive inflammatory infiltrates causing oedema and muscle atrophy [13].

The diagnostic performance of SWE was strong for discriminating IIM from healthy controls in the muscles that showed significant differences. For the best performing muscle (SM), there was a 92% chance of correctly distinguishing IIM from healthy muscle. But the diagnostic performance of BB and stretched quadriceps was not much better than a coin toss. However, it should be noted that an extreme design such as this (health compared with diagnosed IIM) cannot measure clinically relevant diagnostic performance.

These exploratory muscle assessments suggest that IIM patients lost, on average, half of their strength and functional performance compared with healthy controls. The moderate correlations with muscle elasticity were heterogeneous and inconsistent across the different tests and muscles. Abnormal resting muscle elasticity can be the precursor to the commonly observed muscle weakness. It can be hypothesised that the loss in muscle stiffness might limit force transmission capability and may be responsible for diminished muscle strength and functional capacities.

Despite the significant abnormal findings, these preliminary findings indicate that the stretched muscle elasticity was preserved and unaffected by IIM. If confirmed, there are two explanations for this finding. First, the functional stretching property of individual muscle fibres remains intact in IIM [32]. Second, infiltrative inflammatory cells are known to attack passive components, such as the extracellular matrix, which plays a role in providing the resting passive elastic property for muscle [33].

Our results of reduced muscle stiffness match those observed by McCullough et al. in a case–control study of nine active IIM patients using MRE, which also only observed a statistically significant difference in stiffness during the relaxed resting position (*p* = 0.01) [14]. Overall, these trends of altered muscle stiffness support the concept of the detrimental effects of IIM on muscle tissue and its biomechanical property. Although the results can be linked primarily to the IIM pathological processes, secondary confounders and underlying factors cannot be ruled out, such as drug-induced myopathy.

Not all studies have observed a reduction in muscle elasticity. On the contrary, Song et al. [16] compared affected versus normal muscles within each IIM patient and reported that the affected muscles had greater stiffness than adjacent healthy muscles, as indicated by high strain ratios of strain elastography. The discrepancy could be due to the different elastographic modalities used in addition to the potential sampling bias in the placement of the small ROI within the assumed “nearby normal muscle” on ultrasound.

Of particular interest was the normal BB stiffness in IIM patients, which could be attributed to the disease’s relative predilection for the proximal lower thigh and hip girdle muscles [20]. To our knowledge, only one study has been published so far, Bachasson et al. [34] that utilised SWE in myositis, which focused on the BB in inclusion body myositis patients. Although their study design lacked a control group, the researchers reported a significant but weak correlation between BB stiffness and muscle weakness (r_s_ = 0.36, *p* < 0.05).

Our results indicated that MRI signs of activity (i.e. oedema) and damage (i.e. atrophy) were associated with less muscle stiffness in IIM on a number of the muscles investigated. Potential underlying mechanisms include increased intra- and extra-cellular water content in the case of oedema and loss of myofibre contractile properties in atrophy. However, this association is in contrast to the findings of Song et al. [16] and Berko et al. [17], who reported no statistically significant correlation using strain elastography. The technical and mechanical factors of strain elastography may have limited its capability to detect the myositis-induced elasticity changes. We did not find a significant correlation between SWV and MRI scores of fatty infiltration, which is in contrast to previous studies [35, 36]. This could be due to the relatively low prevalence of moderate/severe fatty infiltration in our sample. Overall, the inconsistent SWE–MRI correlations across all muscles could be explained by the variability in MRI muscle involvement depending on IIM subtypes [37].

The failure of muscle elastography to correlate with IIM disease activity was reported by Song et al. [16] and Berko et al. [17]. The latter, however, used the physician’s grading of disease activity as the measure instead of CK. On the other hand, Botar-Jid et al. [15] demonstrated a close graphical proportionality between CK and average hue values of the strain elastography box. This was not substantiated by a statistical test. Previous studies on MRI also failed to correlate with CK [38]. In our study, the lack of correlation could be due to the heterogeneous and wide range of CK values.

Regarding muscles, the VL showed a substantive difference in SWV between the groups and was associated with muscle strength impairment and moderate correlations with MRI. It is a more accessible and easier muscle to scan because of its location and size, and also does not require special positioning like the hamstrings. Therefore, we would recommend that future SWE research uses VL as the most reliable muscle to be assessed.

Our study has several limitations. The study is considered relatively small; nevertheless, the sample size is larger than previous studies on older modalities. The differences between the types of myositis and the effects of the medications including steroid and disease-modifying agents is also unknown. Inter-reader reproducibility for SWE was not analysed owing to the unavailability of a secondary reader. Cut-off values were not computed to calculate sensitivity and specificity owing to the unbefitting study design and small sample size. We did not compare SWE with grey-scale and Doppler findings, as it is not well demonstrated in the literature and not currently used as a routine investigation for myositis. Despite these limitations, we recruited well-matched healthy controls and tested the correlations with strength, CK and MRI to provide an extensive assessment of SWE for the first time.

The results indicate that SWE is a promising non-invasive and quantitative method for detecting active IIM. The relative cost-effectiveness and real-time data are two additional advantages. Our work has revealed the common muscles affected and the best muscle position in which to detect the changes. SWE can be most useful in monitoring disease activity, as several available methods, including biopsy, may not be feasible on a routine basis.

Future studies should evaluate newly suspected and treatment-naive IIM patients with SWE and then follow them prospectively to determine first, if SWE readings predict a future diagnosis of IIM, and second, if SWE is responsive to treatment changes. To achieve a power of 90% (alpha 0.05), the required sample sizes in future studies to detect 20%, 15% and 10% SWV difference in the VL are 50, 80 and 154 participants per group respectively.

In conclusion, thigh muscle stiffness, as quantitatively measured by SWE, appears to be lower in active IIM patients compared with matched healthy controls. Reduced muscle stiffness is likely to be associated with muscle weakness and MRI signs of oedema and atrophy. However, the results did not correlate with fatty infiltration or disease activity (CK level). SWE shows promise as a non-invasive tool for the investigation of biomechanical changes in muscle in patients with IIM. Further studies are needed to confirm these findings and to investigate the responsiveness of SWE for monitoring disease activity.

## Electronic supplementary material

ESM 1 (DOCX 30 kb)

## References

[1] Miller FW (2009). Classification of idiopathic inflammatory myopathies. The inflammatory myopathies.

[2] Ponyi A, Borgulya G, Constantin T, Váncsa A, Gergely L, Dankó K (2005). Functional outcome and quality of life in adult patients with idiopathic inflammatory myositis. Rheumatology.

[3] Cardy CM, Potter T (2007). The predictive value of creatine kinase, EMG and MRI in diagnosing muscle disease. Rheumatology.

[4] Domenichini R, Pialat J-B, Podda A, Aubry S (2017). Ultrasound elastography in tendon pathology: state of the art. Skeletal Radiol.

[5] Dirrichs T, Quack V, Gatz M, Tingart M, Kuhl CK, Schrading S (2016). Shear wave elastography (SWE) for the evaluation of patients with tendinopathies. Acad Radiol.

[6] Aubry S, Nueffer JP, Tanter M, Becce F, Vidal C, Michel F (2015). Viscoelasticity in Achilles tendonopathy: quantitative assessment by using real-time shear-wave elastography. Radiology.

[7] Mathevon L, Michel F, Aubry S, Testa R, Lapole T, Arnaudeau LF (2018). Two-dimensional and shear wave elastography ultrasound: a reliable method to analyse spastic muscles?. Muscle Nerve.

[8] Wu C-H, Ho Y-C, Hsiao M-Y, Chen W-S, Wang T-G (2017). Evaluation of post-stroke spastic muscle stiffness using shear wave ultrasound elastography. Ultrasound Med Biol.

[9] Pichiecchio A, Alessandrino F, Bortolotto C, Cerica A, Rosti C, Raciti MV (2018). Muscle ultrasound elastography and MRI in preschool children with Duchenne muscular dystrophy. Neuromuscul Disord NMD.

[10] Lacourpaille L, Gross R, Hug F, Guével A, Péréon Y, Magot A (2017). Effects of Duchenne muscular dystrophy on muscle stiffness and response to electrically-induced muscle contraction: a 12-month follow-up. Neuromuscul Disord.

[11] Alfuraih AM, O’Connor P, Hensor E, Tan AL, Emery P, Wakefield RJ (2018). The effect of unit, depth, and probe load on the reliability of muscle shear wave elastography: variables affecting reliability of SWE. J Clin Ultrasound.

[12] Alfuraih AM, O’Connor P, Tan AL, Hensor E, Emery P, Wakefield RJ (2017). An investigation into the variability between different shear wave elastography systems in muscle. Med Ultrason.

[13] Kagen LJ (2009). The inflammatory myopathies.

[14] McCullough MB, Domire ZJ, Reed AM, Amin S, Ytterberg SR, Chen Q (2011). Evaluation of muscles affected by myositis using magnetic resonance elastography. Muscle Nerve.

[15] Botar-Jid C, Damian L, Dudea SM, Vasilescu D, Rednic S, Badea R (2010). The contribution of ultrasonography and sonoelastography in assessment of myositis. Med Ultrason.

[16] Song Y, Lee S, Yoo DH, Jang K-S, Bae J (2016). Strain sonoelastography of inflammatory myopathies: comparison with clinical examination, magnetic resonance imaging and pathologic findings. Br J Radiol.

[17] Berko NS, Hay A, Sterba Y, Wahezi D, Levin TL (2015). Efficacy of ultrasound elastography in detecting active myositis in children: can it replace MRI?. Pediatr Radiol.

[18] Julious SA (2005). Sample size of 12 per group rule of thumb for a pilot study. Pharm Stat.

[19] Lancaster GA, Dodd S, Williamson PR (2004). Design and analysis of pilot studies: recommendations for good practice. J Eval Clin Pract.

[20] Dalakas MC, Hohlfeld R (2003). Polymyositis and dermatomyositis. Lancet.

[21] Bohan A, Peter JB (1975). Polymyositis and dermatomyositis. N Engl J Med.

[22] Oddis CV, Reed AM, Aggarwal R, Rider LG, Ascherman DP, Levesque MC (2013). Rituximab in the treatment of refractory adult and juvenile dermatomyositis and adult polymyositis: a randomized, placebo-phase trial. Arthritis Rheum.

[23] Fees E (1992). Grip strength.

[24] Wall JC, Bell C, Campbell S, Davis J (2000). The timed get-up-and-go test revisited: measurement of the component tasks. J Rehabil Res Dev.

[25] Agarwal S, Kiely PDW (2006). Two simple, reliable and valid tests of proximal muscle function, and their application to the management of idiopathic inflammatory myositis. Rheumatology.

[26] Jones CJ, Rikli RE, Beam WC (1999). A 30-s chair-stand test as a measure of lower body strength in community-residing older adults. Res Q Exerc Sport.

[27] Stefanik JJ, Guermazi A, Zhu Y, Zumwalt AC, Gross KD, Clancy M (2011). Quadriceps weakness, patella Alta, and structural features of patellofemoral osteoarthritis. Arthritis Care Res.

[28] Pinal-Fernandez I, Casal-Dominguez M, Carrino JA, Lahouti AH, Basharat P, Albayda J (2017). Thigh muscle MRI in immune-mediated necrotising myopathy: extensive oedema, early muscle damage and role of anti-SRP autoantibodies as a marker of severity. Ann Rheum Dis.

[29] Guimaraes JB, Zanoteli E, Link TM, de Camargo LV, Facchetti L, Nardo L (2017). Sporadic inclusion body myositis: MRI findings and correlation with clinical and functional parameters. AJR Am J Roentgenol.

[30] Cox FM, Reijnierse M, van Rijswijk CS, Wintzen AR, Verschuuren JJ, Badrising UA (2011). Magnetic resonance imaging of skeletal muscles in sporadic inclusion body myositis. Rheumatology.

[31] Sheskin DJ (2003). Handbook of parametric and nonparametric statistical procedures.

[32] Krivickas LS, Amato AA, Krishnan G, Murray AV, Frontera WR (2005). Preservation of in vitro muscle fiber function in dermatomyositis and inclusion body myositis: a single fiber study. Neuromuscul Disord.

[33] De Bleecker JL, De Paepe B, Vanwalleghem IE, Schroder JM (2002). Differential expression of chemokines in inflammatory myopathies. Neurology.

[34] Bachasson D, Dubois GJR, Allenbach Y, Benveniste O, Hogrel J-Y (2018). Muscle shear wave elastography in inclusion body myositis: feasibility, reliability and relationships with muscle impairments. Ultrasound Med Biol.

[35] Rosskopf AB, Ehrmann C, Buck FM, Gerber C, Flück M, Pfirrmann CW (2015). Quantitative shear-wave US elastography of the supraspinatus muscle: reliability of the method and relation to tendon integrity and muscle quality. Radiology.

[36] Gilbert F, Klein D, Weng AM, Köstler H, Schmitz B, Schmalzl J (2017). Supraspinatus muscle elasticity measured with real time shear wave ultrasound elastography correlates with MRI spectroscopic measured amount of fatty degeneration. BMC Musculoskelet Disord.

[37] Maurer B, Walker UA (2015). Role of MRI in diagnosis and management of idiopathic inflammatory myopathies. Curr Rheumatol Rep.

[38] Zheng Y, Liu L, Wang L, Xiao J, Wang Z, Lv H (2015). Magnetic resonance imaging changes of thigh muscles in myopathy with antibodies to signal recognition particle. Rheumatology (Oxford).

