# Supplementary material for: Muscle shear wave elastography in idiopathic inflammatory myopathies: a case–control study with MRI correlation
Source: Skeletal Radiol. 2019 Feb 27;48(8):1209–19. doi: 10.1007/s00256-019-03175-3 (PMC6584706; doi:10.1007/s00256-019-03175-3)
Supplement: Supplementary file 1 — (DOCX 30 kb) [file 256_2019_3175_MOESM1_ESM.docx]

Supplementary material

Table 1. Primary MRI acquisition parameters per sequence.

| Sequence | Voxel size (mm) | FOV (mm) | Resolution (base × phase) | TR (msec) | TE (msec) | Slice thickness (mm) | Flip angle |
| --- | --- | --- | --- | --- | --- | --- | --- |
| t1_tse_COR | 2.0×1.5×3.0 | 480 | 320 × 75% | 658 | 8.8 | 3 | 150° |
| t1_tse_SAG | 2.0×1.5×4.0 | 480 | 320 × 75% | 658 | 8.8 | 4 | 150° |
| t1_tse_AX | 1.2×1.2×5.0 | 300 | 256 × 100% | 850 | 9.1 | 5 | 160° |
| STIR_AX | 1.2×1.2×5.0 | 300 | 256 × 100% | 6550 | 87 | 5 | 140° |
| T2_se_AX_FS | 1.2×1.2×5.0 | 300 | 256 × 100% | 1500 | 9.6 | 5 | 180° |

COR= coronal; SAG= sagittal; AX= axial; TR= Repetition time; TE= Time to echo; tse= turbo spin echo; se= spin echo; STIR= short TI inversion recovery; FS= Fat suppression.

Table 2. Definition and scoring criteria of the evaluated MRI parameters.

| Oedema | Definition | Increased intramuscular signal intensity within muscle tissues on STIR-weighted images. |
| --- | --- | --- |
|  | Scoring | 0 = none.  1 = up to 1/3 of muscle volume involved.  2 = 1/3 – 2/3 of muscle volume involved.  3 = greater than 2/3 of muscle volume involved. |
| Fatty infiltration | Definition | Fatty replacement of muscle tissue, defined as intramuscular T1 hyper intense signal, which suppresses on STIR or fat-saturated images. |
|  | Scoring | 0 = none.  1 = up to 1/3 of muscle volume involved.  2 = 1/3 – 2/3 of muscle volume involved.  3 = greater than 2/3 of muscle volume involved. |
| Atrophy | Definition | Evident reduction of muscle volume based on the subjective assessment of the muscle’s cross-sectional area at the mid-belly, compared to other muscle groups. |
|  | Scoring | 0 = none (no loss of bulk).  1 = up to 1/3 loss of bulk.  2 = 1/3 – 2/3 loss of bulk.  3 = greater than 2/3 loss of bulk. |

**Table 3 Inter-reader agreement for MRI scores of IIM.**

| **Muscle** | **ICC** | **95% CI** |
| --- | --- | --- |
| **Vastus lateralis** | .98 | .96, .99 |
| **Rectus Femoris** | .98 | .95, .99 |
| **Vastus Medialis** | .99 | .95, .99 |
| **Vastus Intermedius** | .96 | .92, .98 |
| **Biceps brachii** | .97 | .93, .98 |
| **Biceps Femoris** | .98 | .95, .99 |
| **Semitendinosus** | .98 | .96, .99 |
| **Semimembranosus** | .95 | .91, .98 |
| **Vastus lateralis-stretched** | .99 | .98, .99 |
| **Rectus Femoris-stretched** | .85 | .68, .92 |
| **Vastus Medialis-stretched** | .94 | .88, .97 |
| **Vastus lateralis-stretched** | .93 | .87, .97 |

**Table 4. SWE correlations with clinical and muscle test variables for the IIM patients.**

|  |  | VL | RF | VM | VI | BF | ST | SM |
| --- | --- | --- | --- | --- | --- | --- | --- | --- |
| Age | **Coeff** | -.31 | -.22 | -.25 | -.18 | -.34 | -.05 | -.34 |
|  | ***p* value** | .15 | .3 | .26 | .42 | .12 | .82 | .13 |
| BMI | **Coeff** | -.08 | -.39 | .21 | **-.52^*^** | -.08 | -.2 | -.19 |
|  | ***p* value** | .72 | .07 | .33 | **.013** | .73 | .38 | .41 |
| Disease duration (months) | **Coeff** | .01 | -.03 | .1 | -.17 | .26 | -.09 | .14 |
|  | ***p* value** | .98 | .91 | .66 | .46 | .24 | .68 | .53 |
| Serum creatinine (IU/L) | **Coeff** | .02 | .13 | .28 | .04 | -.3 | -.34 | -.2 |
|  | ***p* value** | .92 | .56 | .19 | .86 | .17 | .12 | .37 |
| ETGUGT, sit to stand (sec) | **Coeff** | **-.69^**^** | -.12 | -.17 | -.33 | -.32 | -.13 | -.04 |
|  | ***p* value** | **.001** | .63 | .49 | .2 | .22 | .63 | .89 |
| ETGUGT, Gait initiation (sec) | **Coeff** | -.43 | -.34 | .22 | **-.70^**^** | -.44 | -.36 | -.27 |
|  | ***p* value** | .07 | .17 | .39 | **.002** | .08 | .16 | .29 |
| ETGUGT, Walk 1 (sec) | **Coeff** | **-.55^*^** | -.33 | -.02 | **-.70^**^** | -.57^*^ | -.07 | -.37 |
|  | ***p* value** | **.019** | .19 | .93 | **.002** | .017 | .8 | .14 |
| ETGUGT, Turn around (sec) | **Coeff** | **-.49^*^** | -.32 | .01 | **-.60^*^** | -.44 | -.19 | -.27 |
|  | ***p* value** | **.040** | .2 | .95 | **.010** | .08 | .46 | .3 |
| ETGUGT, Walk 2 (sec) | **Coeff** | -.42 | -.36 | .07 | **-.64^**^** | -.45 | .02 | -.3 |
|  | ***p* value** | .09 | .14 | .78 | **.006** | .07 | .93 | .24 |
| ETGUGT, Slow down, stop (sec) | **Coeff** | -.27 | -.19 | .01 | -.4 | -.19 | .02 | -.16 |
|  | ***p* value** | .28 | .46 | .96 | .11 | .46 | .94 | .55 |
| ETGUGT, Total time (sec) | **Coeff** | **-.56^*^** | -.37 | .02 | **-.64^**^** | **-.51^*^** | -.17 | -.32 |
|  | ***p* value** | **.017** | .14 | .94 | **.006** | **.036** | .52 | .21 |
| 30-sec Chair stand test | **Coeff** | **.51^*^** | -.07 | .37 | .14 | .4 | .08 | .31 |
|  | ***p* value** | **.012** | .74 | .08 | .53 | .07 | .73 | .15 |
| Handgrip Strength (kg) | **Coeff** | **.47^*^** | -.02 | .01 | .05 | **.62^**^** | .27 | **.45^*^** |
|  | ***p* value** | **.025** | .94 | .97 | .83 | **.002** | .22 | **.033** |
| Knee extension torque (Nm/kg) | **Coeff** | .33 | -.12 | -.06 | -.02 | **.47^*^** | .32 | .38 |
|  | ***p* value** | .15 | .62 | .79 | .95 | **.034** | .16 | .09 |
| Knee flexion torque (Nm/kg) | **Coeff** | .42 | .11 | .01 | .19 | **.60^**^** | .2 | .31 |
|  | ***p* value** | .06 | .64 | .97 | .42 | **.005** | .4 | .18 |
| Knee extension power (W/kg) | **Coeff** | .33 | -.09 | -.06 | .02 | **.48^*^** | .26 | .36 |
|  | ***p* value** | .14 | .71 | .8 | .94 | **.033** | .26 | .12 |
| Knee flexion power (W/kg) | **Coeff** | .43 | .09 | .12 | .19 | **.53^*^** | .19 | .25 |
|  | ***p* value** | .05 | .71 | .6 | .42 | **.015** | .42 | .3 |

**Coeff= correlation coefficient.**

***. Correlation is significant at the 0.05 level (2-tailed).**

****. Correlation is significant at the 0.01 level (2-tailed).**

**Table 5. MRI muscle characteristics of the IIM patients.**

| **Muscle \| MRI score** | | **Oedema** | | **Fatty infiltration** | | **Atrophy** | |
| --- | --- | --- | --- | --- | --- | --- | --- |
|  |  | Count | % | Count | % | Count | % |
| **Vastus lateralis (VL)** | Normal | 12 | 52.2% | 8 | 34.8% | 15 | 65.2% |
|  | Mild | 5 | 21.7% | 9 | 39.1% | 2 | 8.7% |
|  | Moderate | 0 | 0% | 1 | 4.3% | 2 | 8.7% |
|  | Severe | 6 | 26.1% | 5 | 21.7% | 4 | 17.4% |
| **Rectus Femoris (RF)** | Normal | 13 | 56.5% | 13 | 56.5% | 15 | 65.2% |
|  | Mild | 4 | 17.4% | 7 | 30.4% | 4 | 17.4% |
|  | Moderate | 0 | 0% | 2 | 8.7% | 2 | 8.7% |
|  | Severe | 6 | 26.1% | 1 | 4.3% | 2 | 8.7% |
| **Vastus Medialis (VM)** | Normal | 14 | 60.9% | 12 | 52.2% | 17 | 73.9% |
|  | Mild | 4 | 17.4% | 6 | 26.1% | 2 | 8.7% |
|  | Moderate | 2 | 8.7% | 3 | 13.0% | 3 | 13.0% |
|  | Severe | 3 | 13.0% | 2 | 8.7% | 1 | 4.3% |
| **Vastus Intermedius (VI)** | Normal | 12 | 52.2% | 14 | 60.9% | 16 | 69.6% |
|  | Mild | 3 | 13.0% | 3 | 13.0% | 2 | 8.7% |
|  | Moderate | 3 | 13.0% | 2 | 8.7% | 1 | 4.3% |
|  | Severe | 2 | 21.7% | 4 | 17.4% | 4 | 17.4% |
| **Biceps Femoris (BF)** | Normal | 10 | 43.5% | 6 | 26.1% | 15 | 65.2% |
|  | Mild | 7 | 30.4% | 15 | 65.2% | 6 | 26.1% |
|  | Moderate | 5 | 21.7% | 2 | 8.7% | 1 | 4.3% |
|  | Severe | 1 | 4.3% | 0 | 0% | 1 | 4.3% |
| **Semitendinosus (ST)** | Normal | 13 | 56.5% | 7 | 30.4% | 13 | 56.5% |
|  | Mild | 7 | 30.4% | 10 | 43.5% | 6 | 26.1% |
|  | Moderate | 3 | 13.0% | 5 | 21.7% | 3 | 13.0% |
|  | Severe | 0 | 0% | 1 | 4.3% | 1 | 4.3% |
| **Semimembranosus (SM)** | Normal | 12 | 52.2% | 6 | 26.1% | 13 | 56.5% |
|  | Mild | 7 | 30.4% | 14 | 60.9% | 7 | 30.4% |
|  | Moderate | 3 | 13.0% | 2 | 8.7% | 3 | 13.0% |
|  | Severe | 1 | 4.3% | 1 | 4.3% | 0 | 0% |
